# Case Report: Urgent endovascular treatment of subclavian artery injury after blunt trauma

**DOI:** 10.12688/f1000research.5963.1

**Published:** 2014-12-19

**Authors:** Taka-aki Nakada, Koji Idoguchi, Hiroshi Fukuma, Hidefumi Ono, Shota Nakao, Tetsuya Matsuoka

**Affiliations:** 1 Senshu Trauma and Critical Care Center, Osaka, 598-8577, Japan

**Keywords:** Endovascular treatment, blunt trauma, subclavian artery injury, open clavicle fracture

## Abstract

Subclavian arterial injury is rare and potentially life-threatening, particularly when it leads to arterial occlusion, causing limb ischemia, retrograde thromboembolization and cerebral infarction within hours after injury. Here we report a blunt trauma case with subclavian arterial injury, upper extremity ischemia, and the need for urgent treatment to salvage the limb and prevent cerebral infarction. A 41-year-old man had a left, open, mid-shaft clavicle fracture and left subclavian artery injury accompanied by a weak pulse in the left radial artery, decreased blood pressure of the left arm compared to the right, and left hand numbness. Urgent debridement and irrigation of the open clavicle fracture was followed by angiography for the subclavian artery injury. The left distal subclavian artery had a segmental dissection with a thrombus. Urgent endovascular treatment using a self-expanding nitinol stent successfully restored the blood flow and blood pressure to the left upper extremity. Endovascular treatment is a viable option for cases of subclavian artery injury where there is a risk of extremity ischemia and cerebral infarction.

## Introduction

Subclavian arterial injury caused by blunt trauma is rare with potentially high morbidity and mortality ^1, 2^. Open clavicle fractures caused by blunt trauma are also rare ^3, 4^. Here we report a blunt trauma case with open clavicle fractures and subclavian artery injury accompanied by upper extremity ischemia and the need for urgent treatment.

## Case report

**Figure 1.**
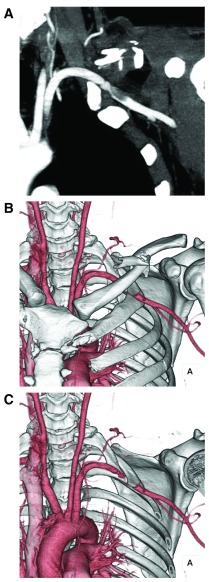
( **A**) Subclavian artery injury shown on contrast-enhanced computed tomography. ( **B** and **C**) Clavicle fractures and subclavian artery injury shown on three-dimensional computed tomography angiography.

**Figure 2.**
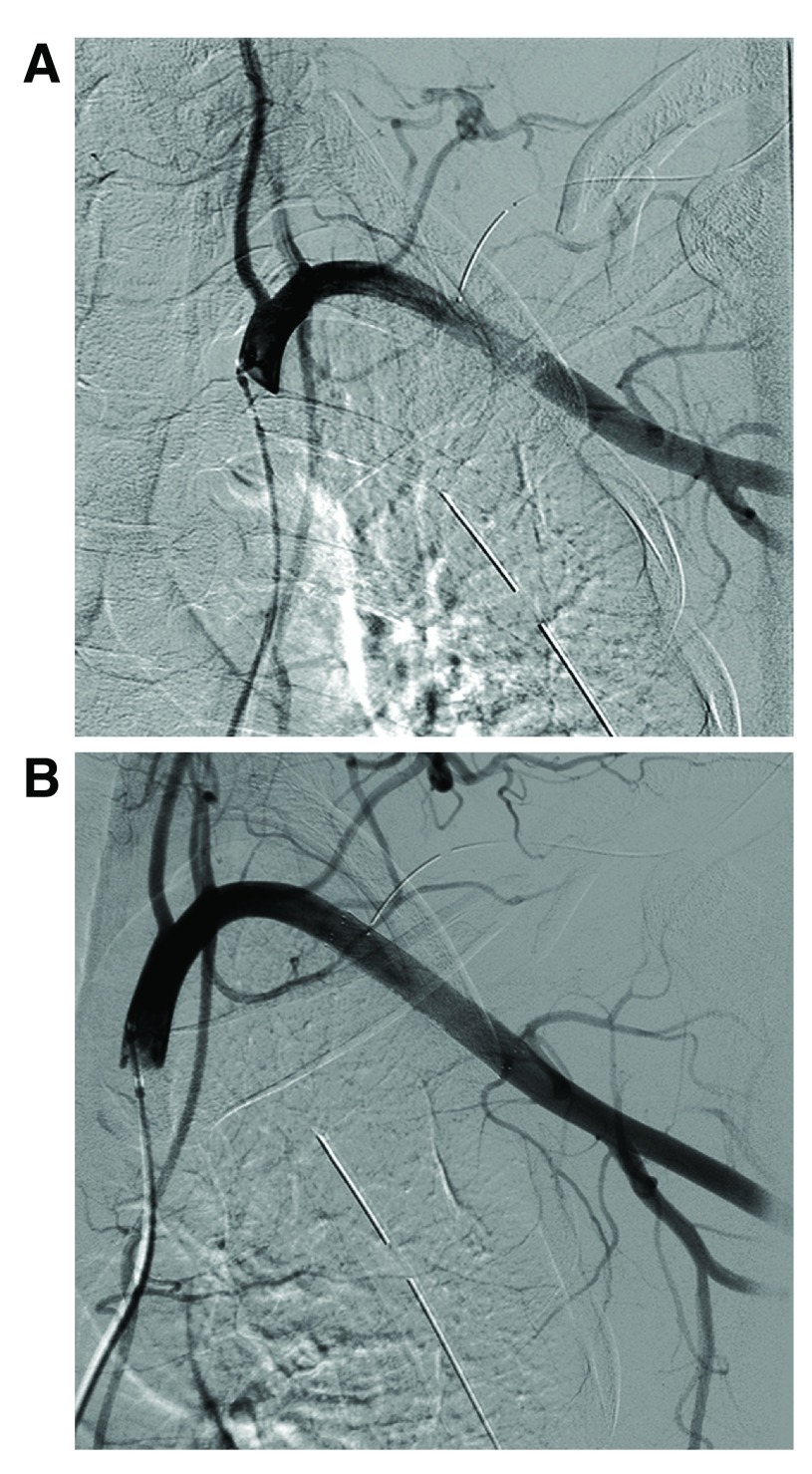
( **A**) Angiography showing segmental dissection of the distal subclavian artery with preserved blood flow to the left upper extremity. ( **B**) Angiography of the subclavian artery showing an adequate stent expansion and restoration of blood flow.

A 41-year-old man, who had no significant previous medical or family history, was thrown from the rear seat of a vehicle during an accident on the motorway. He was transferred to the emergency department of our hospital. Upon admission, he had an open airway, normal breathing with a respiratory rate of 16 breaths/min, was hemodynamically stable with a blood pressure of 123/79 mmHg, and a pulse rate of 88 beats/min. He was conscious and scored E3 for eye opening, V5 for verbal response, and M6 for motor response on the Glasgow Coma Scale. He had a left pneumothorax, a left, open, mid-shaft clavicle fracture accompanied by a 10 mm-sized laceration with numerous subcutaneous air bubbles trapped in the soft tissue on the lateral end of the clavicle, and left subclavian arterial injury (Gustilo Grade I) ( Figure 1A–C). He had multiple lacerations of the forehead without abnormal findings in computed tomography of the head and neck. Both hands were warm with brisk capillary refill in the fingers. The radial and ulnar pulses in the left hand were palpable, but markedly weaker compared to those of the right hand. The blood pressure of the left arm was approximately half that of the right arm blood pressure. Despite no muscle weakness in the upper extremities, the patient had left hand numbness. The Injury Severity Score was 11. The patient was treated with urgent debridement and irrigation for the open clavicle fracture in the operating room followed by urgent angiography for the subclavian artery injury. Initial selective angiography of the left subclavian artery via the right common femoral artery revealed a segmental dissection of the distal subclavian artery with preserved blood flow to the left upper extremity ( Figure 2A). Subsequent intravascular ultrasound via the left brachial artery revealed an intimal flap and a compressed true lumen by a thrombus of the pseudo lumen in the distal subclavian artery (length of the lesion, 3 cm). An 8 mm × 40 mm self-expanding nitinol stent (Smart Control, Cordis) was deployed. Adequate stent expansion and restoration of blood flow of the subclavian artery were confirmed ( Figure 2B). After the endovascular stenting, the left radial and ulnar pulses were remarkably improved and the blood pressure difference between the left and right arm was significantly eliminated. Antithrombotic therapy to prevent stent thrombosis using intravenous heparin targeting aPTT of 2 times the control aPTT for 9 days was followed by an antiplatelet therapy using aspirin 100 mg plus cilostazol 200 mg daily for 12 months. On day 6, an open reduction and internal fixation of the clavicle fracture using a Kirschner wire were performed. The patient was discharged on day 22 and continued to be free of complications at the 2-month follow-up with stent patency determined using color duplex ultrasonography.

## Discussion

Clavicle fractures are common injuries and mostly treated non-operatively with good outcomes, while open clavicle fractures due to blunt trauma are rare, accounting for 0.2–1.3% of all clavicle fractures in a trauma clinic or Level I trauma center ^3, 4^. Open clavicle fractures caused by penetrating trauma are frequently associated with a great vessel injury, including subclavian artery injury, compared to those caused by blunt trauma ^3^.

Subclavian artery injuries through blunt trauma are rare with a reported incidence of less than 1% of all arterial injuries or thoracic traumatic injuries ^5– 7^. Subclavian artery injuries are caused by stretching, transection, or compression of the subclavian artery by broken bone fragments. Unexpected neurovascular symptoms, a pseudoaneurysm rupture, or a thrombus associated with upper extremity ischemia often initiate weeks or months after initial injury. There have been reports of patients who had delayed symptom recognition but were treated successfully in late phases ^8, 9^. However, there have been cases with massive hemorrhage due to transection of the subclavian artery ^1^ or cerebral infarction due to occlusion of the subclavian artery ^2^ within hours after injury, highlighting the importance of urgent therapeutic management of subclavian artery injury. Our case presented an intimal injury of the subclavian artery with a thrombus leading to upper extremity ischemia, which could cause retrograde thromboembolization and cerebral infarction. We thus urgently treated for prevention of cerebral infarction and to salvage the limb.

An open surgical approach is one treatment option for subclavian artery injury. However, this approach requires an extensive incision to obtain proximal and distal control, which is invasive, difficult to perform, and associated with high morbidity ^6, 10^. In our case, the patient had an open fracture, which is a risk factor for graft infection in vascular surgery. Advances in endovascular treatments for vascular injuries have achieved increasing success for treatment of subclavian artery injury caused by penetrating trauma such as a gunshot, stab, or iatrogenic catheter injury ^10^. Endovascular treatment is a viable option for cases of subclavian artery injury where there is a risk of extremity ischemia and cerebral infarction.

## Consent

Written informed consent for publication of clinical details and images was obtained from the patient.
