# Supplementary material for: Case Report: Urgent endovascular treatment of subclavian artery injury after blunt trauma
Source: F1000Res. 2014 Dec 19;3:310. [Version 1] doi: 10.12688/f1000research.5963.1 (PMC4329670; doi:10.12688/f1000research.5963.1)
Supplement: Peer review history

# Peer review: Referee response for version 1

**DOI:** 10.5256/f1000research.6380.r7279

**Published:** 2015-01-14

**Authors:** Tal Hörer

**Affiliations:** 1 Department of Cardio-Thoracic and Vascular Surgery, Örebro University Hospital and Örebro University, Örebro, Sweden

I would like to congratulate the authors for the successful treatment and report. This is a report about endovascular trauma treatment and in my eyes, state of the art for modern trauma surgery.

I would humbly ask that the authors look at the English as there are some minor English corrections that can be done.

I have read this submission. I believe that I have an appropriate level of expertise to confirm that it is of an acceptable scientific standard.

# Peer review: Referee response for version 1

**DOI:** 10.5256/f1000research.6380.r7100

**Published:** 2015-01-14

**Authors:** Urvi Shukla

**Affiliations:** 1 Department of Critical Care, Aditya Birla Memorial Hospital, Pune, Maharashtra, India

First of all, let me congratulate the team on a case report that is well written and a patient that was well managed. This report again highlights the fact that displaced clavicular fractures can lead to vascular complications, either thrombotic or bleed. These complications may lead to greater morbidity or even a life threatening event.  It needs a high index of suspicion to recognize these injuries, more so because timely intervention may save someone's limb or life. The authors have recognized the injury early and managed it aptly. Endovascular repair of vessel injury is now standard of care with sophisticated stents and procedural advances. Subclavian artery dissection with thrombosis in the false lumen has been managed as per evidence base.

I have read this submission. I believe that I have an appropriate level of expertise to confirm that it is of an acceptable scientific standard.
